# Plants’ Impact on the Human Brain—Exploring the Neuroprotective and Neurotoxic Potential of Plants

**DOI:** 10.3390/ph17101339

**Published:** 2024-10-07

**Authors:** Georgiana Moise, Alex-Robert Jîjie, Elena-Alina Moacă, Iasmina-Alexandra Predescu, Cristina Adriana Dehelean, Alina Hegheș, Daliborca Cristina Vlad, Roxana Popescu, Cristian Sebastian Vlad

**Affiliations:** 1 Department of Clinical Pharmacology, The Doctoral School of Medicine, “Pius Brînzeu” County Emergency Clinical Hospital Timisoara, “Victor Babeș” University of Medicine and Pharmacy, 2nd Eftimie Murgu Square, 300041 Timisoara, Romania; georgiana.moise@umft.ro; 2 Department of Toxicology, Drug Industry, Management and Legislation, Faculty of Pharmacy, “Victor Babeș” University of Medicine and Pharmacy, 2nd Eftimie Murgu Square, 300041 Timisoara, Romania; alex-robert.jijie@umft.ro (A.-R.J.); alina.moaca@umft.ro (E.-A.M.); iasmina-alexandra.predescu@umft.ro (I.-A.P.); cadehelean@umft.ro (C.A.D.); 3 Research Centre for Pharmaco-Toxicological Evaluation, Faculty of Pharmacy, “Victor Babeș” University of Medicine and Pharmacy, 2nd Eftimie Murgu Square, 300041 Timisoara, Romania; 4 Department II—Pharmaceutical Technology, Faculty of Pharmacy, “Victor Babeș” University of Medicine and Pharmacy, 2nd Eftimie Murgu Square, 300041 Timisoara, Romania; 5 Formulation and Technology of Drugs Research Center, Faculty of Pharmacy, “Victor Babeș” University of Medicine and Pharmacy, 2nd Eftimie Murgu Square, 300041 Timisoara, Romania; 6 Department IV—Department of Biochemistry and Pharmacology, Division of Pharmacology, Faculty of Medicine, “Victor Babeș” University of Medicine and Pharmacy, 2nd Eftimie Murgu Square, 300041 Timisoara, Romania; vlad.daliborca@umft.ro (D.C.V.); vlad.cristian@umft.ro (C.S.V.); 7 Centre for Molecular Research in Nephrology and Vascular Disease, Faculty of Medicine, “Victor Babeș” University of Medicine and Pharmacy, 2nd Eftimie Murgu Square, 300041 Timisoara, Romania; popescu.roxana@umft.ro; 8 Department II—Department of Microscopic Morphology, Division of Cell and Molecular Biology II, Faculty of Medicine, “Victor Babeș” University of Medicine and Pharmacy, 2nd Eftimie Murgu Square, 300041 Timisoara, Romania

**Keywords:** neurotoxicity, neuroprotection, neurotoxic plants, neuroprotective plants, brain, neurological properties of plants, nervous system, medicinal plants, toxic plants, neurological health

## Abstract

Background: Plants have long been recognized for their potential to influence neurological health, with both neuroprotective and neurotoxic properties. This review explores the dual nature of plant-derived compounds and their impact on the human brain. Discussion: Numerous studies have highlighted the neuroprotective effects of various phytoconstituents, such as those found in *Ginkgo biloba*, *Centella asiatica*, *Panax ginseng*, *Withania somnifera*, and *Curcuma longa*. The neuroprotective compounds have demonstrated antioxidant, anti-inflammatory, and cognitive-enhancing properties, making them promising candidates for combating neurodegenerative diseases and improving brain function. Polyphenolic compounds, triterpenic acids, and specific phytocompounds like the ones from EGb 761 extract have shown interactions with key enzymes and receptors in the brain, leading to neuroprotective outcomes. However, this review also acknowledges the neurotoxic potential of certain plants, such as the *Veratrum* species, which contains steroidal alkaloids that can cause DNA damage and disrupt neurological function, or *Atropa belladonna*, which interfere with the normal functioning of the cholinergic system in the body, leading to a range of symptoms associated with anticholinergic toxicity. Conslusions: This review also emphasizes the need for further research to elucidate the complex mechanisms underlying the neuroprotective and neurotoxic effects of plant-derived compounds, as well as to identify novel phytoconstituents with therapeutic potential. Understanding the complex relationship between plants and the human brain is crucial for harnessing the benefits of neuroprotective compounds while mitigating the risks associated with neurotoxic substances. This review provides a comprehensive overview of the knowledge on the neurological properties of plants and highlights the importance of continued research in this field for the development of novel therapeutic strategies targeting brain health and neurological disorders.

## 1. Introduction

Neurodegenerative diseases are a major challenge to global health, with a higher probability of occurrence in the elderly. These pathologies irreversibly affect neurons, affecting the quality of life of people and leading to a reduced life expectancy [1].

Plants have been intertwined with human civilization since ancient times, offering sustenance, healing, and cultural significance. However, the relationship between plants and the human brain remains relatively unexplored, rich with promise and danger. The brain, a marvel of complexity and fragility, is susceptible to degeneration over time, making the exploration of neuroprotective and neurotoxic compounds from plants a matter of essential importance [2]. The human brain, with its intricate network of neurons and neurotransmitters, requires meticulous care to function optimally, and plants, with their diverse array of bioactive phytoconstituents, possess the capacity to either bolster or undermine its performance [3]. The significance of investigating plants with neurological effects lies in the potential discovery of novel neuroprotective agents that could combat neurodegenerative diseases and enhance cognitive function [4]. Plants have been found to possess compounds that can protect neurons from damage caused by neurotoxins, reduce neuroinflammation, and even improve memory and cognitive abilities [5].

Numerous plant-derived compounds have been identified as powerful neuroprotective agents, offering a shield against neurological disorders and bolstering cognitive function [6]. They contribute to the strengthening of the human system and combat the various processes of nerve cell damage, thus supporting the proper functioning of the brain and stimulating brain regeneration [7]. For instance, studies have highlighted the neuroprotective properties of compounds found in plants like *Hypericum perforatum*, commonly known as St. John’s Wort, which have shown promise in mitigating neurotoxicity and potentially benefiting individuals with conditions like Alzheimer’s and Parkinson’s disease [8,9]. Moreover, plants like *Melissa officinalis*, *Matricaria recutita*, and *Cymbopogon citratus* have been studied for their antioxidant effects, which play a crucial role in mitigating oxidative stress in the brain. Oxidative stress is implicated in the pathogenesis of various neurological disorders, and the presence of antioxidants in these plants showcases their potential neuroprotective benefits. By scavenging free radicals and reducing lipid peroxidation, plant extracts can help maintain neuronal health and function, highlighting the dual role of plants in neurological outcomes [10]. Phytocompounds derived from medicinal herbs and dietary sources play a crucial role in maintaining the chemical balance of the brain by influencing the function of neurotransmitter receptors [11]. These compounds, such as polyphenolic compounds found in fruits, vegetables, herbs, and nuts, exhibit neuroprotective properties that can enhance memory and cognitive function [12]. Reactive oxygen species are responsible for most pathologies, which can be neutralized with plant polyphenolic compounds, which protect cellular functions [13]. Triterpenic acids, a class of phytocompounds with diverse therapeutic properties, including neuroprotective effects, have been extensively studied for their potential to address various health issues, including neurodegenerative diseases [14]. Plants like *Gentiana olivieri* contain phytocompounds such as gentiopicroside and isoorientin, which have demonstrated anti-inflammatory and antioxidant properties, contributing to their neuroprotective effects [15]. As oxidative stress is followed by lipid peroxidation, compounds with antioxidant action offer better protection against oxidative stress and damage [16]. Additionally, the activation of SIRT1 by resveratrol, a polyphenolic compound found in red grapes, has shown promising neuroprotective effects against oxidative stress and toxicity induced by amyloid-β peptides [17]. Studies have also emphasized the neuroprotective efficacy of phytoconstituents from plants like *Calligonum polygonoides*, with compounds such as apigenin triacetate showing interactions with key enzymes and receptors in the brain, leading to improvements in conditions like hypercholesterolemia and neurodegenerative changes in brain regions like the cortex and hippocampus [18].

Conversely, some plants contain neurotoxic substances that can disrupt the delicate balance of the brain, leading to cognitive decline, neurological damage, and in severe cases, life-threatening consequences [19]. For instance, the *Veratrum* species contains steroidal alkaloids that have been reported to cause DNA damage and decrease blood pressure in vivo. These alkaloids can have detrimental effects on the brain and overall neurological function [20]. Similarly, aconitine, a diterpenoid alkaloid found in *Aconitum* species, is known for its anti-inflammatory and analgesic properties but can also induce severe arrhythmia and neurotoxicity. The presence of aconitine highlights how certain plant compounds can have dual effects, including neurotoxic outcomes [21]. Moreover, plants like *Lathyrus* species contain β-N-oxalyl-L-α,β-diaminopropionic acid (BOAA), an acute neurotoxic agent that can lead to lathyrism, a neurological disorder associated with the consumption of these plants. The neurotoxic compound in *Lathyrus* plants is species-specific and has been linked to the development of neurolathyrism in individuals consuming these plants over extended periods [22,23]. Additionally, pyrrolizidine alkaloids (PAs), a group of secondary metabolites found in various plants, have been studied for their pharmacological applications but also pose risks due to their potential neurotoxic effects [24]. These examples underscore the diverse range of plants containing neurotoxic compounds that can adversely affect neurological health.

Furthermore, the impact of plant chemicals on neurological systems extends to interactions with neurotransmission pathways. Aconitine, for instance, disrupts serotonin neurotransmission via 5-hydroxytryptamine receptors in zebrafish embryos, indicating a direct effect on synaptic transmission. This disruption in neurotransmission highlights how plant-derived compounds can interfere with essential signaling processes in the brain, leading to neurotoxic outcomes [25]. Additionally, the presence of certain alkaloids in plants can have indirect neurotoxic effects. For example, some mushrooms and plants contain compounds like dencichine and cyanogens, which can exhibit neurotoxicity in humans due to their interaction with metal elements in the environment. This indirect neurotoxic potential emphasizes the complex interplay between plant chemicals, environmental factors, and neurological health [26].

The relationship between dosage and the neurotoxic or neuroprotective effects of plants is a fundamental principle in toxicology, famously articulated by Paracelsus: “The dose makes the poison” (Latin: “Sola dosis facit venenum”) [27,28]. This adage underscores that any substance can be both beneficial and harmful, depending on its concentration. Numerous studies have documented that various plant extracts exhibit neuroprotective properties at certain dosages while potentially being neurotoxic at others. For instance, compounds derived from plants have been shown to provide neuroprotection against neurodegenerative diseases when administered in appropriate amounts [29]. Conversely, excessive exposure to these same compounds can lead to adverse effects, highlighting the critical role of dosage in determining their therapeutic value [30]. Thus, understanding the dose–response relationship is essential for harnessing the therapeutic potential of plants while mitigating their risks [31].

This exploration of the dual nature of plants’ impact on the human brain aims to illuminate the intricate interplay between plants and neurological health. By unraveling the complexities of this relationship, we can harness the therapeutic potential of plants to enhance brain function while remaining vigilant against the risks plants may pose. Understanding the mechanisms underlying the neuropharmacological activities of the phytocompounds is crucial for unlocking their full therapeutic potential and advancing novel strategies for brain health and disease management.

The scope of this article extends to elucidating both the beneficial and detrimental effects of some plant species on the human brain, underscoring the critical need for a nuanced understanding of neurology for exploiting herbal medicine effectively. Through a comprehensive analysis of neuroactive compounds in different plant species, this article aims to emphasize the importance of using nature’s resources to improve neurological well-being. The findings presented in this article are poised to have far-reaching implications across diverse fields, from medicine and neuroscience to agriculture and environmental science, fostering a deeper understanding of the profound influence that plants wield over the human brain.

## 2. Neurotoxic Plants

### 2.1. Defining Neurotoxicity and Some Mechanisms of Plant-Induced Neurotoxicity

Neurotoxicity, in the context of plant-induced neurotoxicity, refers to the harmful effects that certain plants or their bioactive components can have on the nervous system. This phenomenon involves various mechanisms that can lead to damage or dysfunction in the nervous system, impacting both the peripheral and central nervous systems. Several studies have delved into the mechanisms underlying neurotoxicity caused by different plant-derived substances, shedding light on the intricate processes involved [32,33,34].

The mechanisms of plant-induced neurotoxicity are multifaceted and involve various pathways that can lead to detrimental effects on neuronal cells. One such mechanism involves the production of neurotoxic compounds that can disrupt normal neuronal function and lead to neurodegeneration. For example, bufadienolides found in some succulent plants from the Crassulaceae family (like *Cotyledon*, *Tylecodon*, or *Kalanchoe*) have been identified as neurotoxic substances that can induce damage to the nervous system. These compounds can interfere with mitochondrial function, leading to oxidative stress, neuroinflammation, and ultimately apoptosis, contributing to neurotoxicity [35]. Moreover, the neurotoxicity induced by certain plant-derived substances can manifest in various ways, affecting different aspects of neurological function. For instance, cardiac glycosides have been shown to produce marked neurotoxicity when administered intracerebroventricularly in animal models, highlighting the potent effects these compounds can have on the nervous system [34]. Certain plant-derived substances, such as alkaloids, have been shown to affect sodium channels in the nervous system, leading to neurotoxic effects. By targeting essential components of neuronal signaling, these compounds can disrupt the normal functioning of neurons and potentially cause damage to the brain [36]. Additionally, some plants contain phytocompounds that can induce neurotoxicity through mechanisms such as oxidative stress, mitochondrial dysfunction, and inflammation. These compounds may interfere with critical processes in the nervous system, ultimately resulting in neuronal damage and dysfunction [37].

In the context of neurodegenerative diseases, the role of mitochondria in generating reactive oxygen species (ROS) has been implicated in the pathogenesis of conditions like Parkinson’s and Alzheimer’s diseases [38]. Plant compounds that interfere with mitochondrial function or exacerbate ROS production can contribute to neuronal damage and accelerate the progression of neurodegenerative disorders. Moreover, the disruption of endoplasmic reticulum–mitochondria interactions by certain plant-derived substances can lead to neurotoxicity, as seen in the case of phthalates causing morphological changes and cognitive impairments in the brain [39,40].

Overall, some plants can exert brain-damaging effects and negatively impact the nervous system through the production of neurotoxic compounds that interfere with essential neuronal processes involving a variety of pathways that can lead to neuronal damage and cell death. From disrupting mitochondrial function and inducing oxidative stress to altering neurotransmitter levels and promoting neuroinflammation, some plant-derived substances can have diverse mechanisms of neurotoxicity. Understanding these mechanisms is important for identifying potential neurotoxic plants and developing strategies to mitigate their harmful effects.

### 2.2. Common Neurotoxic Plants—Mechanisms of Neurotoxicity, Symptoms of Neurotoxicity, Management of Neurotoxic Plant Poisoning

#### 2.2.1. *Aconitum* Species (Monkshood)

##### Historical Background

*Aconitum* species, belonging to the Ranunculaceae family, have a long history of use in both Western and Eastern medicine for their pharmacological properties [41]. These plants are known for their rich content of diterpene alkaloids and flavonoids [42]. *Aconitum* alkaloids, particularly aconitine, are considered the characteristic bioactive ingredients of these species and are widely used in traditional medicine. Aconitine has been studied for its therapeutic potential in various conditions such as heart failure, myocardial infarction, neuroinflammatory diseases, rheumatic diseases, and tumors [43]. However, despite their medicinal uses, *Aconitum* species contain highly toxic compounds, especially in their tubers and flowers, which can have detrimental effects on the nervous system and cardiovascular system [44]. *Aconitum* presents a narrow therapeutic window, making it both beneficial and potentially lethal. Therapeutically, the doses of *Aconitum* preparations typically range from 1.5 to 3 g of the raw plant material, depending on the specific formulation and processing method [45,46,47]. However, the toxic and lethal doses are alarmingly close; the minimum lethal dose of aconitine, a principal alkaloid, is reported to be between 2 and 6 mg in humans [48,49]. Accidental overdoses can lead to severe symptoms such as bradycardia, hypotension, and potentially fatal arrhythmias, often resulting in death within 24 h due to cardiovascular collapse [50,51,52]. The therapeutic and toxic effects are compounded by the fact that improper processing of *Aconitum* can significantly increase its toxicity [45,53]. Therefore, careful dosage and processing are critical to mitigate the risks associated with *Aconitum* use.

##### Relevant Toxic Chemical Compounds, Mechanism of Toxicity

The main toxic compound found in *Aconitum* species is aconitine, a diester C19-diterpenoid alkaloid with potent neurotoxic effects [54]. Aconitine has been implicated in inducing cardiotoxicity, neurotoxicity, and mitochondrial damage in various studies [55]. It is known to affect the central nervous system and cardiovascular system, leading to symptoms such as polymorphic arrhythmias, neurotoxicity, hypotension, gastrointestinal disorders, paralysis, and even death [56,57]. The neurotoxic effects of aconitine have been linked to its ability to interfere with cellular signaling pathways, including the TNFα-NLRP3 signaling axis and mitophagy [55]. Additionally, aconitine has been shown to induce P-glycoprotein expression, potentially leading to drug–drug interactions and altered drug metabolism [58]. Another underlying mechanism of toxicity is based on the binding of aconitine to voltage-dependent sodium channels, favoring the influx of Na^+^ ions, concomitant with the increase in intracellular calcium via the Na^+^-Ca^2+^ exchange system [59]. Another diterpenoid alkaloid responsible for neurotoxicity is mesaconitine (MA), manifesting its damaging effects on neuronal cells via autophagy pathways [60].

##### Detoxification Process for Toxic Chemical Compounds, Mechanism of Toxicity

Studies have highlighted the importance of processing and detoxifying *Aconitum* species before use to mitigate their toxic effects [61]. Traditional methods like Śodhana in Ayurveda have been employed for detoxification and modification of the therapeutic activities of poisonous medicinal plants, including *Aconitum* species. The traditional Ayurvedic method of Śodhana involves a series of purification processes aimed at detoxifying toxic plants by reducing their toxicity and enhancing their therapeutic efficacy through the use of specific media such as cow’s milk, which facilitate the transformation of harmful constituents into less toxic forms [62,63,64,65]. This method not only purifies the physical and chemical properties of the plants but also minimizes side effects, thereby making them suitable for therapeutic applications [66,67,68].

These practices aim to reduce the concentration of toxic compounds, particularly alkaloids, which are responsible for the cardiotoxic and neurotoxic effects of *Aconitum* plants [62].

##### Poisoning

In cases of monkshood poisoning, which have been reported in various regions, the symptoms often involve neurological manifestations due to the toxic effects on the nervous system. The combination of *Aconitum* alkaloids with other substances like alcohol can exacerbate the toxicity, leading to central nervous system depression and neurological symptoms [69]. Management of neurotoxic plant poisoning involves supportive care, decontamination, and symptomatic treatment to address the effects on the nervous system and other affected organs [49]. The patient’s vital functions must be constantly checked, especially ventricular arrhythmias which can be treated with amiodarone and flecainide, which has been scientifically proven [51,70].

##### General Conclusion

Overall, *Aconitum* species, known for their medicinal properties, contain potent neurotoxic compounds such as aconitine that can harm the nervous system. Understanding the mechanisms of neurotoxicity, symptoms of poisoning, and appropriate management strategies is crucial in dealing with cases of *Aconitum* poisoning. Processing and detoxification methods play a significant role in reducing the toxic effects of *Aconitum* plants, emphasizing the importance of traditional practices in ensuring the safe use of these potent botanicals.

#### 2.2.2. *Atropa belladonna* (Deadly Nightshade)

##### Historical Background

*Atropa belladonna*, commonly known as deadly nightshade, belongs to the Solanaceae family and has a long history of use in medicine due to its pharmacological properties. This plant contains a variety of alkaloids, with atropine and scopolamine being the most significant, responsible for its anticholinergic effects [71]. These alkaloids, along with hyoscyamine, exert both medicinal and toxic effects on the central and peripheral nervous systems [72]. Consumption of different parts of the *Atropa belladonna* plant, including the fruits, leaves, and roots, can lead to intoxication due to the presence of poisonous tropane alkaloids. Therapeutically, low doses of *Atropa belladonna* can be effective for conditions like asthma and gastrointestinal disorders due to its anticholinergic properties [73,74]. However, the line between therapeutic and toxic doses is narrow; even small amounts can lead to severe poisoning, particularly in children, where doses as low as 10–20 berries can be fatal [75,76]. For instance, atropine is utilized in doses as low as 0.5 to 1 mg for specific medical applications, such as treating bradycardia and as a pre-anesthetic agent [75]. Additionally, homeopathic preparations, such as those diluted to D3, contain minimal alkaloid concentrations, which may still provide therapeutic effects without significant toxicity [77].

##### Relevant Toxic Chemical Compounds, Mechanism of Toxicity

The chemical composition of *Atropa belladonna*, as analyzed in various studies, reveals the presence of tropane alkaloids, including hyoscyamine, atropine, and scopolamine, which contribute to its pharmacological properties. These alkaloids play a significant role in the plant’s toxic effects on the nervous system, leading to symptoms of poisoning when consumed [71,78]. The high alkaloid content in different parts of the *Atropa belladonna* plant underscores the potential dangers associated with its ingestion and the importance of recognizing and managing cases of poisoning effectively [78]. The neurotoxic effects of *Atropa belladonna* are primarily attributed to its alkaloid content, particularly atropine, and scopolamine, which act as anticholinergic agents [72]. These alkaloids interfere with the normal functioning of the cholinergic system in the body, leading to a range of symptoms associated with anticholinergic toxicity.

##### Poisoning

*Atropa belladonna* intoxication clinically manifests as anticholinergic syndrome, characterized by symptoms such as dry mouth, blurred vision, tachycardia, and altered mental status. Additionally, laboratory tests may show abnormalities reflecting the impact of the plant’s toxins on the body [78]. The hallucinogenic, sedative, and antiemetic effects are due to atropine’s ability to inhibit muscarinic acetylcholine receptors in the central nervous system, while the adverse effects of pupil dilation, decreased heart rate, and bronchial constriction occur as a result of blocking nerve impulses from the parasympathetic nervous system [79]. The management of *Atropa belladonna* poisoning involves various strategies to address the symptoms and effects of alkaloid toxicity. In cases of intoxication, especially in children where even small amounts can be fatal, prompt medical intervention is crucial [76]. Physostigmine, an acetylcholinesterase inhibitor, has been used in the treatment of *Atropa belladonna* poisoning to counteract the anticholinergic effects of the plant’s alkaloids. This antidote helps restore cholinergic activity in the body, mitigating the toxic effects of *Atropa belladonna* alkaloids on the nervous system [73].

##### General Conclusion

*Atropa belladonna* poses significant neurotoxic effects due to the presence of alkaloids, which exert anticholinergic actions on the nervous system. The plant’s historical uses in medicine are overshadowed by its potential for toxicity, leading to symptoms of poisoning when consumed. Management of *Atropa belladonna* poisoning involves prompt medical intervention, with physostigmine being used as an antidote to counteract the plant’s toxic effects. Understanding the neurotoxic implications of *Atropa belladonna* alkaloids is crucial for addressing their impact on the nervous system and potential implications for neurological disorders.

#### 2.2.3. *Conium maculatum* (Hemlock)

##### Historical Background

*Conium maculatum*, commonly known as poison hemlock, belongs to the Apiaceae family and is notorious for its high toxicity due to the presence of piperidine alkaloids in its aerial parts [80]. The most significant alkaloid found in *Conium maculatum* is coniine, which can be fatal in doses as low as 150 mg, leading to neurotoxic effects, acute rhabdomyolysis, and acute renal failure [81]. Ingestion of larger amounts can be lethal, as evidenced by historical accounts and clinical cases [82]. Historically, *Conium maculatum* has been used in medicine for various purposes. It has been reported to possess antispasmodic properties and has been used to alleviate conditions such as epilepsy, asthma, angina, rheumatism, and tetanus [83]. Additionally, the plant has been utilized in traditional and alternative medicine systems, including homeopathy, for treating glandular enlargements and cancerous tumors in various parts of the body [84]. The plant’s toxic nature has also been recognized for its acute toxicity to humans and domestic animals globally [85].

##### Relevant Toxic Chemical Compounds, Mechanism of Toxicity

The chemical composition of *Conium maculatum* includes a range of secondary metabolites, such as alkaloids, flavonoids, coumarins, polyacetylenes, and non-volatile oils [80]. Among these compounds, alkaloids have been identified as bioactive constituents in the plant. The presence of alkaloids like coniine in *Conium maculatum* has been linked to its toxic effects on the nervous system and overall neurotoxicity [86]. Other alkaloids present in the plant include N-methylconiine, conhydrine, pseudoconhydrine, and γ-coniceine [87]. These alkaloids, particularly γ-coniceine and coniine, are responsible for the plant’s toxicity and are known to have stereoselective potencies and relative toxicities [88]. The neurotoxic effects of *Conium maculatum*, particularly attributed to coniine, are mediated through its interaction with nicotinic acetylcholine receptors (nAChRs). Coniine acts as an agonist of nAChRs, leading to the activation of these receptors and subsequent effects on muscle movement and function. Studies have shown that coniine can activate fetal muscle-type nAChRs and inhibit fetal movement, highlighting its potent neurotoxic properties [89].

##### Poisoning

Symptoms of neurotoxicity or brain damage resulting from *Conium maculatum* poisoning may include respiratory failure, muscle weakness, paralysis, tremors, seizures, and ultimately death in severe cases. The neurotoxic effects of the plant’s alkaloids can manifest as progressive paralysis starting from the lower extremities and moving upwards, eventually affecting respiratory muscles and leading to respiratory failure. Management of neurotoxic plant poisoning, such as that caused by *Conium maculatum*, involves supportive care and symptomatic treatment. In cases of ingestion or exposure to the plant, it is crucial to seek immediate medical attention. Treatment may include respiratory support, administration of activated charcoal to limit further absorption of toxins, intravenous fluids for hydration, and in severe cases, the use of antidotes if available. Close monitoring of vital signs, neurological status, and respiratory function is essential in managing cases of *Conium maculatum* poisoning [81].

##### General Conclusion

*Conium maculatum*, with its potent neurotoxic alkaloids like coniine, poses a significant risk to human health. The plant’s historical uses in medicine, chemical composition rich in toxic alkaloids, mechanisms of neurotoxicity through nAChR activation, and symptoms of neurotoxicity, and the management of plant poisoning, highlight the importance of understanding and addressing the dangers associated with this toxic plant. Effective management of *Conium maculatum* poisoning requires prompt medical intervention and supportive care to mitigate the potentially severe consequences of neurotoxicity and brain damage resulting from exposure to this plant.

#### 2.2.4. *Oenanthe crocata* (Hemlock Water-Dropwort)

##### Historical Background

*Oenanthe crocata*, commonly known as hemlock water-dropwort, belongs to the Apiaceae family and is recognized for its potent neurotoxic effects [90]. The inhibition of GABA receptors by polyacetylenes extracted from *Oenanthe fistulosa*, closely related to *Oenanthe crocata*, further supports the mechanism through which these compounds affect the nervous system [91].

##### Relevant Toxic Chemical Compounds, Mechanism of Toxicity

This plant contains neurotoxins such as oenanthotoxin, dihydrooenanthotoxin, and cicutoxin, which have been identified as the compounds responsible for its toxic properties [92]. These neurotoxins have been found to block GABAergic responses, leading to symptoms such as facial muscle paralysis, which can manifest as a strained smile [93]. The chemical composition of *Oenanthe crocata* includes polyacetylenes, which have been shown to have inhibitory effects on GABA receptors [91]. Additionally, the essential oil of *Oenanthe crocata* has been found to contain compounds like sabinene, which exhibit antioxidant, antifungal, and anti-inflammatory activities [94]. These chemical constituents play a role in the plant’s overall toxicity and its impact on the nervous system. Furthermore, the presence of the convulsant polyacetylene toxin oenanthotoxin in water hemlock, a plant closely related to *Oenanthe crocata*, highlights the shared neurotoxic properties within the genus [95].

##### Poisoning

Symptoms of neurotoxicity or brain damage resulting from *Oenanthe crocata* poisoning can include autonomic dysfunction, heart issues, and potentially fatal outcomes such as heart arrest. The reversible nature of autonomic dysfunction in *Oenanthe crocata* poisoning suggests that prompt management and intervention can lead to recovery in affected individuals [96]. Moreover, the potent neurotoxic effects of *Oenanthe crocata*, attributed to compounds like oenanthotoxin and dihydrooenanthotoxin, underscore the importance of understanding the plant’s toxic properties and implementing appropriate treatment strategies in cases of poisoning [92]. Management of neurotoxic plant poisoning, such as that caused by *Oenanthe crocata*, involves supportive care to address the symptoms presented by the affected individual. This may include measures to stabilize vital signs, manage any cardiovascular complications, and provide symptomatic treatment to alleviate the effects of neurotoxins on the body. Additionally, in cases of severe poisoning, interventions such as decontamination and administration of antidotes may be necessary to counteract the toxic effects of the plant compounds and prevent further damage to the nervous system [96].

##### General Conclusion

*Oenanthe crocata* poses significant risks due to its neurotoxic effects, primarily mediated by compounds like oenanthotoxin and dihydrooenanthotoxin. These substances interfere with GABAergic responses, leading to symptoms such as facial muscle paralysis. Understanding the chemical composition, mechanisms of neurotoxicity, symptoms of poisoning, and appropriate management strategies is crucial in dealing with cases of *Oenanthe crocata* poisoning and mitigating its damaging effects on the nervous system. Vigilance in recognizing and treating plant poisonings, particularly those involving potent neurotoxins like the ones from *Oenanthe crocata*, is essential to ensure favorable outcomes for affected individuals.

#### 2.2.5. *Ricinus communis* (Castor Plant)

##### Historical Background

*Ricinus communis*, commonly known as the castor oil plant, belongs to the Euphorbiaceae family and has a long history of medicinal use [97]. The neurotoxic effects of *Ricinus communis* have been studied extensively, particularly focusing on its impact on the nervous system [98].

##### Relevant Toxic Chemical Compounds, Mechanism of Toxicity

The chemical composition of *Ricinus communis* plays a crucial role in its neurotoxicity. The plant contains various bioactive compounds such as ricinoleic acid, tannins, flavonoids, cardiac glycosides, steroids, saponins, alkaloids, phlorotannins, and terpenoids, which have been associated with various toxic effects, including neurotoxicity [99,100]. One of the most notorious components of *Ricinus communis* is ricin, a potent toxin attributed to the lectin ricin. Ricin, in particular, has been extensively studied for its ability to inhibit protein synthesis, leading to cell death and tissue damage [101]. Ricin is a 60 to 65 kDa glycoprotein that acts as a lectin and belongs to the A-B toxin family. The lectin ricin, derived from *Ricinus communis*, shares sequence homology with ribosome-inactivating proteins, further elucidating its mechanism of action in disrupting cellular processes [102]. *Ricinus communis* has been found to have neurotoxic effects due to compounds like ricin, which can lead to severe damage to the nervous system. The neurotoxic effects of *Ricinus communis* are attributed to its agglutinin-II component, which has been shown to have detrimental impacts on neural tissues [103].

##### Poisoning

Symptoms of neurotoxicity or brain damage from *Ricinus communis* poisoning may include nausea, vomiting, abdominal pain, diarrhea, seizures, and, in severe cases, respiratory failure and death. The toxic effects on the nervous system can manifest as neurological symptoms such as confusion, dizziness, and muscle weakness. Additionally, exposure to *Ricinus communis* can lead to organ damage, particularly affecting the liver and kidneys, further exacerbating the toxic effects on the body. Management of neurotoxic plant poisoning, such as that caused by *Ricinus communis*, involves supportive care and specific treatments to counteract the effects of the toxins. In cases of ingestion, decontamination methods like gastric lavage or administration of activated charcoal may be employed to reduce further absorption of the toxins. Symptomatic treatment to address specific symptoms such as seizures or respiratory distress is crucial in managing cases of neurotoxicity from plant poisoning. In severe cases, antidotes or specific therapies targeting the toxins’ mechanisms of action may be considered to mitigate the toxic effects on the nervous system [101].

##### General Conclusion

*Ricinus communis*, with its complex chemical composition containing toxic compounds like ricin, poses significant risks to the nervous system and overall health. The plant’s neurotoxic effects are well documented, highlighting the importance of understanding its mechanisms of action, symptoms of toxicity, and appropriate management strategies in cases of poisoning. Further research into the specific pathways through which *Ricinus communis* exerts its neurotoxic effects is essential for developing targeted interventions to mitigate its damaging impacts on the nervous system.

**Figure 1:**
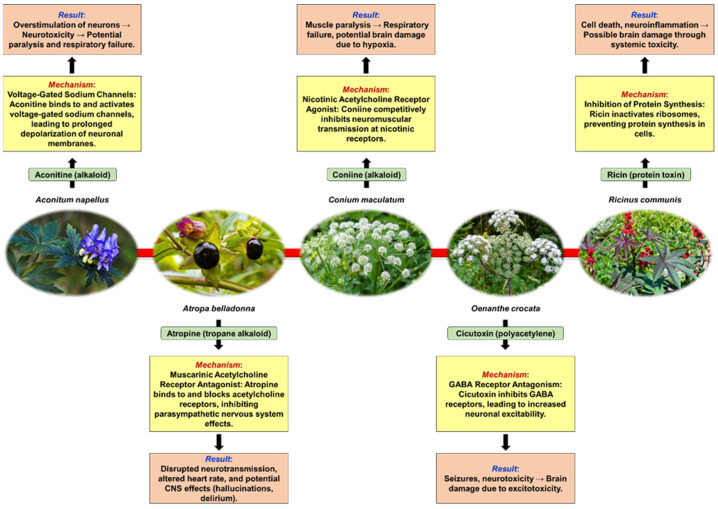
Mechanism of action of some neurotoxic plants: *Aconitum napellus* (https://www.alamy.com/stock-photo/aconitum.html?sortBy=relevant, accessed on 7 August 2024), *Atropa belladonna* (https://www.alamy.com/stock-photo/atropa-belladonna.html?sortBy=relevant, accessed on 7 August 2024), *Conium maculatum* (https://www.alamy.com/stock-photo/conium-maculatum.html?sortBy=relevant, accessed on 7 August 2024), *Oenanthe crocata* (https://www.alamy.com/stock-photo/Oenanthe-crocata.html?sortBy=relevant, accessed on 7 August 2024), and *Ricinus communis* (https://www.alamy.com/stock-photo/ricinus-communis.html?sortBy=relevant, accessed on 7 August 2024).

Figure 1 illustrates the mechanisms of action of some neurotoxic plants, including the Latin names of the plants, their major toxic compounds, the specific mechanisms of neurotoxicity, and the resultant effects on the nervous system. This overview provides insights into how these plants exert their neurotoxic effects, aiding in understanding the potential risks associated with exposure to these botanical sources of toxicity.

Table 1 systematically categorizes various neurotoxic plants, detailing their respective used parts and the toxic secondary metabolites identified within them. Each entry elucidates the mechanism of toxicity associated with the metabolites, providing insight into how these compounds exert their neurotoxic effects. The table further distinguishes between in vivo models utilized in the studies, specifying the lethal dosages administered in each case. The outcomes of the toxicity assessments are succinctly summarized, highlighting the effects observed in the experimental models. This comprehensive overview serves as a valuable resource for understanding the neurotoxic potential of these plants and the underlying biological mechanisms involved.

**Table 1:** Overview of neurotoxic plants: used parts, toxic secondary metabolites, in vivo studies and toxic dosage, and outcomes.

Plant, Family	Used Parts	Toxic Secondary Metabolites	In Vivo Studies and Toxic Dosage		Outcomes	Refs.
*Aconitum napellus* (monkshood), Ranunculaceae	Roots, leaves	diterpenoid alkaloids: aconitine, mesaconitine, hypaconitine, benzoylaconitine, benzoylmesaconine, benzoylhypaconine, lappaconitine, jesaconitine, songorine, heteratisine, napelline	Aconitine	Mouse: 1.8 mg/kg (p.o.), 0.24–0.55 mg/kg (s.c.), 0.28–0.34 mg/kg (i.p.), 0.12 mg/kg (i.v.)	*Aconitum* poisoning disrupts the neural transmission pathway, preventing normal neuronal signals from traversing the synapse, leading to abnormal limb control, paralysis, dizziness, speech difficulties, muscle weakness, twitching, and spasms. Furthermore, the toxic compounds can inflict damage on the central nervous system, potentially causing both temporary and long-lasting neurological disorders, and in severe cases, this condition may culminate in shock or coma.	[51,104]
				Rat: 0.102- 0.112 mg/kg (i.v.)		
				Cat: 0.07–0.13 mg/kg (i.v.)		
				Dog: 0.5 mg/kg (i.v.)		
				Human: 2–5 mg (p.o.)		
			Mesaconitine	Mouse: 1.9 mg/kg (p.o.), 0.19–0.25 mg/kg (s.c.), 0.20–0.23 mg/kg (i.p.), 0.068–0.10 mg/kg (i.v.)		
				Rat: 0.158 mg/kg (i.v.)		
			Hypaconitine	Mouse: 5.8 mg/kg (p.o.), 1.1–1.9 mg/kg (s.c.), 1.0–1.2 mg/kg (i.p.), 0.47 mg/kg (i.v.)		
				Rat: 0.291 mg/kg (i.v.)		
			Benzoylaconine	Mouse: 70 mg/kg (i.p.), 23 mg/kg (i.v.)		
				Rat: > 20.2 mg/kg (i.v.)		
			Benzoylmesaconine	Mouse: 810 mg/kg (p.o.), 230 mg/kg (s.c.), 240 mg/kg (i.p.), 21 mg/kg (i.v.)		
				Rat: > 18.7 mg/kg (i.v.)		
			Benzoylhypaconine	Mouse: 830 mg/kg (p.o.), 130 mg/kg (s.c.), 120 mg/kg (i.p.), 23 mg/kg (i.v.)		
				Rat: > 20.4 mg/kg (i.v.)		
			Lappaconitine	Mouse: 5.9–11.5 mg/kg (i.v.)		
			Jesaconitine	Mouse: 0.23 mg/kg (s.c.)		
			Songorine	Mouse: 106 mg/kg (i.v.)		
			Heteratisine	Mouse: 147 mg/kg (i.v.)		
			Napelline	Mouse: > 147 mg/kg (i.v.)		
*Atropa belladonna* (deadly nightshade), Solanaceae	Leaves, roots, berries	tropane alkaloids: hyoscyamine, atropine, scopolamine	The lethal quantity: >2–4 mg/day Children = 2–5 berries with 2 mg of atropine each (0.2 mg/kg of atropine) Adults = 10–20 berries with 2 mg of atropine each		The anti-muscarinic effects of *Atropa belladonna* poisoning result in a range of symptoms, including confusion, incomprehensible speech, delirium, lethargy, coma, palpitations, flushing, mydriasis, blurred vision, and hallucinations. The anticholinergic properties of these compounds inhibit the action of acetylcholine at muscarinic receptors, leading to the aforementioned neurological and cardiovascular disturbances.	[81,105,106]
*Conium maculatum* (hemlock), Apiaceae	Leaves, seeds	piperidine alkaloids: coniine, γ-coniceine	Rat: >50 mg/kg (p.o.)		*Conium maculatum* poisoning presents with a sequence of clinical manifestations, beginning with heightened activity of motor nerve endings and the central nervous system. This initial stimulation is succeeded by paralysis and a state of depression, leading to impaired movement characterized by slowness and weakness. Subsequently, affected individuals may experience a rapid pulse, hyperventilation, increased urination, respiratory paralysis, and ultimately progress to coma and death. In the initial phase of poisoning, symptoms such as ataxia and headache are evident, whereas heightened salivation, rapid heart rate, and dilation of the pupils arise as a result of the plant’s impact on the autonomic ganglia.	[107,108,109]
			Coniine	Mouse: 100 mg/kg (p.o.), 19 mg/kg (i.v.); 80 mg/kg (s.c.)		
			γ-coniceine	Mouse: 12 mg/kg (p.o.), 2.6 mg/kg (i.v.); 12 mg/kg (s.c.)		
*Oenanthe crocata* (hemlock water-dropwort), Apiaceae	Tubers, leaves, seeds	polyacetylenes: oenanthotoxin, dihydrooenanthotoxin, cicutoxin	Mouse: 17 mg/kg for tubers, 1320 mg/kg for green seeds Goat: 1–2 fresh small tubers or 1 larger tuber, 0.25 g/kg BW (p.o.)		The toxicity of *Oenanthe crocata* is associated with the induction of respiratory paralysis and convulsions, characterized by seizures that occur alongside a depression of motor functions within the central nervous system. This dual manifestation highlights the compound’s detrimental effects on both respiratory and neurological systems.	[110,111,112]
*Ricinus communis* (castor plant), Euphorbiaceae	Seeds, leaves	ricin	Mouse: 30 mg/kg (p.o.), 22 mg/kg (i.p.); 2–10 mg/kg (i.v.), 3.5 µg crude ricin/kg BW (intranasal)—24 μg/kg (injection or inhalation), 20–30 mg/kg (seed ingestion) Rat: 20–30 mg/kg (i.p.) Human: 3–10 μg/kg BW (inhalation—solid or liquid particles), 1–10 μg/kg BW (injection into muscle or vein), 1–20 mg ricin/kg BW = equivalent to approximately 8 seeds (p.o.) Adults: 10–20 seeds Children: 1–6 seeds		Ricin exerts significant neurotoxic effects primarily through its mechanism of action as an inhibitor of protein synthesis. Upon exposure, ricin can lead to a range of neurological symptoms, including seizures, ataxia, and altered mental status. The toxin disrupts cellular function by enzymatically inactivating ribosomal RNA, which impairs protein synthesis and ultimately results in cell death, particularly in neurons. Additionally, ricin can induce inflammation and oxidative stress within the central nervous system, contributing to neuronal damage. The severity of these neurotoxic effects is dose-dependent, with higher exposures leading to more pronounced neurological deficits and potential respiratory failure due to central respiratory depression.	[113,114,115]

p.o. = per os; s.c. = subcutaneously; i.p. = intraperitoneally; i.v. = intravenously.

Table 2 presents the chemical structures of primary secondary metabolites identified in various neurotoxic plants, presenting their structural diversity and highlighting their significance in neurotoxicity.

## 3. Neuroprotective Plants

### 3.1. Defining Neuroprotection and Some Mechanisms of Plant-Induced Neuroprotection

Neuroprotection, in the context of plant-induced effects, refers to the ability of certain compounds derived from medicinal plants and herbal extracts to shield neurons from damage or degeneration, thereby preserving their structure and function. These neuroprotective effects are crucial in combating various neurodegenerative conditions such as Parkinson’s disease (PD), Alzheimer’s disease (AD), stroke, and traumatic brain injuries [116,117,118].

Plants exhibit neuroprotective effects through various mechanisms that involve combating neuroinflammation and oxidative stress and correcting neurotransmitter imbalances. These mechanisms are crucial in maintaining neurological health and protecting against neurodegenerative disorders. Medicinal plants have been found to inhibit the accumulation of protein-based deposits, reduce oxidative stress, alleviate neuroinflammation, and address neurotransmitter deficiencies like acetylcholine and dopamine [119,120,121]. Phytochemicals play a significant role in these neuroprotective effects by acting as antioxidants, anti-inflammatories, anti-apoptotic agents, and inhibitors of enzymes like acetylcholinesterase and monoamine oxidase. Additionally, they exhibit neurotrophic activities that support the growth and survival of neurons [120].

Various plant extracts target different pathways associated with neurodegenerative diseases to provide neuroprotection. These pathways include inhibiting enzymes responsible for neurotransmitter degradation, reducing oxidative stress, preventing amyloid plaque formation, and enhancing mitochondrial function [121]. The neuroprotective effects of plants extend to central nervous system injuries, where compounds derived from plants have shown promise in modulating inflammatory responses, preserving tissues, and aiding in the recovery of motor and cognitive functions. This highlights the diverse ways in which plant-derived compounds can support neuronal health and function [122].

Compounds like ellagic acid, found in fruits and nuts, have been shown to protect dopamine neurons by inhibiting inflammatory processes in microglia [123]. Similarly, flavonoids like quercetin possess free radical scavenging abilities that contribute to neuroprotection by modulating intracellular signals to promote cellular survival [124].

One of the well-studied plant compounds with neuroprotective effects is resveratrol, a compound present in red grapes, known for its neuroprotective potential in conditions like stroke and traumatic brain injury [117]. Moreover, natural products like formononetin, a type of isoflavonoid, have shown promise in preventing and treating neurological diseases due to their beneficial biological activities [125]. These plant-derived compounds act through various pathways to protect neurons from damage, enhance cognitive function, and promote overall brain health [126].

The neuroprotective effects of plant compounds extend beyond traditional medicinal herbs to include fruits like *Schisandra*, which contain lignans known for their antioxidant and neuroprotective properties [127,128]. Additionally, extracts from plants such as *Astragalus* and *Rhodiola* have been found to protect against neurodegeneration and enhance cognitive function [129,130].

Furthermore, the neuroprotective properties of plant-derived compounds are not limited to specific conditions but have broad implications for overall brain health and function. For example, compounds like salidroside, found in *Rhodiola* plants, exhibit neuroprotective effects by reducing oxidative stress and promoting cell survival [129,131]. Similarly, the aqueous extract of *Areca catechu* has shown significant neuroprotective activity against H_2_O_2_-induced cell death in neurons, highlighting the potential of plant-based interventions in preventing neuronal damage [132].

Phenolic compounds found in plants have been extensively studied for their neuroprotective effects. Compounds like resveratrol, curcumin, apocynin, and epi-gallocatechin have shown significant neuroprotective properties. These compounds, derived from sources like grapes, *Curcuma longa*, *Picrorhiza kurroa*, and *Camellia sinensis*, exhibit antioxidant properties that are beneficial in conditions such as Alzheimer’s disease, Parkinson’s disease, and stroke [133]. Moreover, dietary plant polyphenols have been identified as safe agents with potent neuroprotective effects against various neurodegenerative diseases. These findings underscore the potential of plant-based polyphenols in supporting brain health and combating neurodegeneration [134]. Polyphenolic compounds, particularly flavonoids, have been identified as potent neuroprotective agents that offer protection against neonatal hypoxic–ischemic brain injury [135]. Interestingly, some plant extracts have demonstrated neuroprotective effects by mitigating neurotoxicity-induced damage in neuronal cells. For instance, extracts from plants like *Centella asiatica*, *Panax ginseng*, and *Gastrodia elata* have shown neuroprotective properties in cell culture models, suggesting a potential therapeutic role in combating neurotoxicity [4,136]. The neuroprotective activities of asiaticoside, madecassoside, and other plant compounds further demonstrate the therapeutic properties of plant-derived agents in supporting neurological health [137].

The diverse mechanisms of action of plant-derived compounds make them promising candidates for neuroprotection. For instance, neuroprotective phenylpropanoids like caffeic acid have been shown to reverse neurotoxicity induced by amyloid (A)β, highlighting their potential to mitigate neurodegenerative processes [138]. Additionally, pyranocoumarins from *Angelica gigas* demonstrate neuroprotective effects through activities such as free radical scavenging and modulation of apoptotic pathways [139,140]. *Cannabis sativa* contains compounds like Delta9-tetrahydrocannabinol (THC) and cannabidiol (CBD) that possess anti-neuroinflammatory and neuroprotective activities [141]. Furthermore, compounds from natural sources, such as zonarol from brown algae, activate pathways like Nrf2/ARE to provide neuroprotection. The activation of such pathways is crucial in enhancing cellular defense mechanisms against oxidative stress and promoting neuronal survival [142]. The neuroprotective effects of compounds like polyprenylated tetraoxygenated xanthones from *Hypericum monogynum* roots highlight the diverse sources of neuroprotective agents found in plants [143].

Limonene, a compound found in various plants, has been shown to counteract neurotoxicity triggered by Aβ1–42 oligomers, highlighting its neuroprotective potential [144]. Similarly, *Syzygium* species have been found to exert neuroprotective effects by modulating various pathways such as cholinergic transmission, inflammation, oxidative stress, and antioxidant enzyme activity [145].

Plant-derived neuroprotective agents act through multiple mechanisms to interrupt the cycle of cell death and promote neuronal survival [146]. Compounds like carvacrol and p-cymene exhibit anti-enzymatic properties and neuroprotective effects against oxidative stress, emphasizing their potential in preserving neuronal function [147]. Flavonoids and diarylheptanoids, important phenolic compounds in plants, play a crucial role in imparting neuroprotective effects through their antioxidant and anti-inflammatory properties [148]. The neuroprotective potentials of extracts from the Amaryllidaceae plant family and their alkaloids further highlight the diverse sources of neuroprotective agents found in nature [149].

Phytoestrogens have been studied for their neuroprotective and neurotrophic efficacy in cultured hippocampal neurons, showcasing their potential to promote cell survival and protect against neurotoxic insults [150].

These examples collectively illustrate the vast array of plant-derived compounds that exhibit neuroprotective effects through various mechanisms, highlighting their potential in preventing and managing neurodegenerative disorders.

### 3.2. Common Neuroprotective Plants—Mechanisms of Neuroprotection, Neurological Benefits, Clinical Applications, and Research Findings

#### 3.2.1. *Ginkgo biloba* (Ginkgo)

##### Historical Background

*Ginkgo biloba*, a plant from the Ginkgoaceae family, has a long history of use in traditional medicine and is known for its neuroprotective effects. The neuroprotective properties of *Ginkgo biloba* are attributed to various mechanisms of action, including antioxidation, anti-inflammation, anti-apoptosis, defense against mitochondrial dysfunction, modulation of tau protein phosphorylation, ion homeostasis, and the induction of growth factors. These mechanisms collectively contribute to the plant’s ability to protect neurons from damage and degeneration, making it a promising candidate for the treatment of neurodegenerative diseases [151].

##### Neuroprotective Chemical Metabolites

The chemical composition of Ginkgo biloba plays a crucial role in mediating its neuroprotective effects. The plant contains bioactive compounds such as flavonoids and terpenes, including ginkgolides and bilobalides, which contribute to its pharmacological properties. These compounds exert various actions, such as antioxidant, anti-inflammatory, and neuroprotective effects, which collectively enhance the plant’s therapeutic potential in neurological disorders [152].

##### Neuroprotective Properties

Research has shown that *Ginkgo biloba* extract, particularly the special extract EGb 761, demonstrates neuroprotective effects in neurodegenerative diseases with multifactorial origins [153]. Studies have highlighted the plant’s potential in mitigating Alzheimer’s disease-like pathologies, such as amyloid-β accumulation and neuronal loss, suggesting that *Ginkgo biloba* extract could serve as a prophylactic target for Alzheimer’s disease [154]. Additionally, *Ginkgo biloba* has been reported to reverse amyloid β-peptide-induced isoprostane production in the brain, emphasizing its role in ameliorating neurodegenerative changes associated with aging through its antioxidant effects [155].

##### Clinical Applications

The neuroprotective effects of *Ginkgo biloba* extend beyond Alzheimer’s disease, with studies indicating its potential in treating various neurological conditions. For instance, *Ginkgo biloba* has been found to have beneficial effects on memory and cognitive function in healthy older individuals and patients with dementia [156]. Furthermore, the plant has shown promise in treating attention-deficit disorder and other neuropsychiatric symptoms [157]. These findings underscore the diverse neurological benefits of *Ginkgo biloba* and its potential applications in enhancing brain health and function.

In addition to its neurological benefits, *Ginkgo biloba* has been investigated for its cardiovascular and renal protective effects. Studies suggest that the plant may have cardioprotective properties, including antioxidant, antiplatelet, antithrombotic, and vasodilatory effects, which could contribute to its potential in managing hypertension and related cardiovascular conditions [158]. Moreover, *Ginkgo biloba* extract has been identified as an effective antioxidant and free radical scavenger, highlighting its nephroprotective potential in combating oxidative stress-induced renal damage [159].

Clinical applications of *Ginkgo biloba* in the management of neurodegenerative diseases, cognitive impairment, and other neurological conditions have been supported by research findings. Meta-analyses and systematic reviews have synthesized the efficacy and safety of *Ginkgo biloba* extract in treating dementia, including Alzheimer’s disease and vascular dementia. These studies have highlighted the plant’s ability to improve cognitive function, memory, and overall brain health, making it a valuable therapeutic option for individuals with neurological disorders [160].

##### General Conclusion

*Ginkgo biloba* stands out as a natural remedy with significant neuroprotective effects attributed to its diverse mechanisms of action and bioactive compounds. From its historical uses in traditional medicine to its current applications in clinical practice, *Ginkgo biloba* continues to be a subject of interest in neuroscience research. The plant’s ability to safeguard neurons, enhance cognitive function, and protect against neurodegenerative processes underscores its potential as a valuable asset in the field of neuroprotection and neurological health.

#### 3.2.2. *Centella asiatica* (Gotu Kola)

##### Historical Background

*Centella asiatica*, commonly known as gotu kola, belongs to the Apiaceae family and has a long history of traditional medicinal use [161]. This herb has gained recognition for its diverse pharmacological properties, including anti-inflammatory, antioxidant, and neuroprotective effects [162]. The neuroprotective potential of *Centella asiatica* has been a subject of interest in scientific research due to its ability to mitigate oxidative stress, inflammation, and cellular damage in various conditions [163]. The plant’s chemical composition, rich in triterpenes like asiaticoside and madecassoside, flavonoids, and other bioactive compounds, contributes to its therapeutic effects [164].

##### Neuroprotective Chemical Metabolites

*Centella asiatica* stands out as a botanical agent with profound neuroprotective effects attributed to its antioxidant, anti-inflammatory, and anti-apoptotic properties. The plant’s rich chemical composition, including triterpenes and flavonoids, contributes to its therapeutic potential in safeguarding neuronal health and function. From mitigating oxidative stress and inflammation to supporting nerve regeneration and cognitive function, *Centella asiatica* offers a holistic approach to neurological well-being. Further research and clinical trials are warranted to fully elucidate the mechanisms of action and optimize the therapeutic use of this botanical marvel in neurological disorders and neurodegenerative conditions [165].

##### Neuroprotective Properties

Studies have demonstrated that *Centella asiatica* exhibits significant neuroprotective effects through various mechanisms. One such mechanism involves its ability to modulate oxidative stress by enhancing antioxidant defenses and reducing lipid peroxidation [165]. Additionally, *Centella asiatica* has been shown to regulate inflammatory mediators, such as interleukins and tumor necrosis factor, thereby attenuating inflammation-induced neuronal damage [166]. The herb’s constituents, particularly asiaticoside, have been found to inhibit cell death pathways, including the expression of caspase-3, in the brain, highlighting its anti-apoptotic properties [167].

##### Clinical Applications

The neuroprotective benefits of *Centella asiatica* extend to neurological disorders and conditions. Research has indicated its potential to ameliorate neurological dysfunction under hypoxic conditions, suggesting a protective role in oxygen-deprived environments [168]. Furthermore, *Centella asiatica* has shown promise in addressing cognitive disorders, neurodegenerative diseases like Alzheimer’s and Parkinson’s, and even peripheral nerve injuries [169,170]. The herb’s ability to promote nerve regeneration and support neurodifferentiation of mesenchymal stem cells underscores its therapeutic versatility in neurological health [170].

##### General Conclusion

Clinical applications of *Centella asiatica* in neuroprotection have been explored in various contexts. Studies have highlighted its efficacy in reducing oxidative stress and improving cognitive function, making it a potential candidate for managing age-related cognitive decline and memory impairment [171]. Furthermore, the herb has demonstrated anti-seizure properties, suggesting a role in managing conditions like epilepsy [172]. *Centella asiatica*’s ability to enhance neuronal differentiation and protect against neurotoxic insults positions it as a valuable natural remedy for promoting brain health and resilience [173].

#### 3.2.3. *Panax ginseng* (Ginseng)

##### Historical Background

*Panax ginseng*, a perennial plant from the Araliaceae family, has a rich history of medicinal use dating back thousands of years in traditional Chinese medicine as a tonic and prophylactic agent [174,175]. Modern research has explored the health benefits of *Panax ginseng*, revealing its potential neuroprotective effects. The plant contains bioactive compounds known as ginsenosides, which have been linked to many of its therapeutic properties [176]. Studies have demonstrated that these ginsenosides play a crucial role in the neuroprotective mechanisms of *Panax ginseng* [177].

##### Neuroprotective Chemical Metabolites

The chemical composition of *Panax ginseng*, particularly its ginsenosides, plays a remarkable role in its diverse therapeutic effects. These bioactive compounds have been extensively studied for their pharmacological activities, including their neuroprotective mechanisms [178]. The ginsenosides present in *Panax ginseng* have been linked to its ability to improve cognitive function, protect against neurodegeneration, and alleviate symptoms of various neurological disorders [174].

##### Neuroprotective Properties

The neuroprotective effects of *Panax ginseng* are attributed to its ability to scavenge free radicals and induce enzymes like superoxide dismutase and glutathione peroxidase, which help protect against oxidative stress and neuronal damage [177]. Additionally, *Panax ginseng* has been found to modulate the RAGE/NF-κB pathway, reducing oxidative stress and inflammation in the brain and thereby improving cognitive deficits induced by advanced glycation end products [178]. These mechanisms contribute to the plant’s overall neuroprotective properties.

##### Clinical Applications

Neurodegenerative diseases, such as Alzheimer’s, Parkinson’s, and Huntington’s diseases, present significant challenges in healthcare. Research suggests that *Panax ginseng* and its bioactive components show promise in the treatment and prevention of these conditions [176]. Ginsenoside Rb1, a key active ingredient in *Panax ginseng*, has been specifically highlighted for its neuroprotective mechanisms in various central nervous system diseases [175]. This compound showcases the potential of *Panax ginseng* in mitigating neurological damage and related pathologies [174].

Clinical studies have further supported the neurological benefits of *Panax ginseng*. Research indicates that *Panax ginseng* supplementation can enhance cognitive performance in patients with Alzheimer’s disease [179]. The plant’s neuroprotective effects extend beyond cognitive enhancement to include the prevention and treatment of various neurodegenerative diseases [176]. Moreover, *Panax ginseng* has been found to protect against mercury-induced neurotoxicity, highlighting its potential to mitigate the harmful effects of heavy metal exposure on the central nervous system [180].

In addition to its neuroprotective effects, *Panax ginseng* exhibits a wide range of pharmacological activities. The plant has been reported to have anti-inflammatory, antioxidative, and anti-fibrotic properties, making it beneficial for various organ systems, including the liver, lung, heart, and kidney [181]. Furthermore, *Panax ginseng* has been shown to enhance muscle recruitment, reduce perceived effort during exercise, and accelerate muscle force recovery in athletes, indicating its potential in sports medicine and performance enhancement [182].

##### General Conclusion

*Panax ginseng*, with its rich history in traditional medicine and extensive modern research backing its therapeutic properties, stands out as a promising natural remedy with significant neuroprotective effects. The plant’s bioactive compounds, particularly ginsenosides, contribute to its diverse pharmacological activities, including its ability to protect against neurodegenerative diseases, enhance cognitive function, and mitigate neuronal damage. Clinical studies have provided evidence of *Panax ginseng*’s efficacy in improving neurological health, making it a valuable candidate for the prevention and treatment of various central nervous system disorders.

#### 3.2.4. *Withania somnifera* (Ashwagandha)

##### Historical Background

*Withania somnifera*, commonly known as ashwagandha, belongs to the Solanaceae family and has a rich historical background in traditional medicine systems like Ayurveda. This plant has gained significant attention due to its diverse pharmacological properties, including its neuroprotective effects. Studies have highlighted the potential of *Withania somnifera* in protecting against neurological disorders through its bioactive components, particularly withanolide A, which has shown neuroprotective properties against hypoxia-induced and cerebral ischemia-induced apoptosis [183]. The neuroprotective effects of *Withania somnifera* have been demonstrated in various experimental models, such as in MPTP-intoxicated mice, where the plant extract increased dopamine content in the striatum and attenuated Parkinsonism [184].

##### Neuroprotective Chemical Metabolites

The wide chemical composition of *Withania somnifera* contributes to its neuroprotective mechanisms. Withanolides, alkaloids, steroidal lactones, and glycosides present in the plant play a crucial role in its pharmacological activities. These bioactive compounds exhibit antioxidant, anti-inflammatory, and immunomodulatory properties, which are essential for neuroprotection [185]. *Withania somnifera* has been reported to act as an antioxidant, protecting against lipid peroxidation and oxidative stress, thereby safeguarding erythrocytes from damage [186]. Additionally, the plant’s ability to modulate pathways like JAK-STAT and MAP kinase signaling further enhances its neuroprotective potential [187].

##### Clinical Applications

The neuroprotective benefits of *Withania somnifera* extend to clinical applications, where it has been explored as an adjunctive treatment for conditions like Parkinson’s disease. Studies have shown that *Withania somnifera* can improve motor symptoms in Parkinson’s patients due to its neuroprotective effects, leading to enhanced dopamine activity [188]. Furthermore, the plant has been investigated for its effects on cognitive and psychomotor performance in healthy individuals, indicating its potential to support overall brain health and function [189].

##### Neuroprotective Properties

Research findings have also highlighted the role of *Withania somnifera* in combating age-related neurodegenerative diseases. The plant’s protective role in inflammaging, a chronic low-grade inflammatory state associated with aging, has been discussed, emphasizing its potential to preserve brain health and function during the aging process. *Withania somnifera*’s ability to mitigate oxidative stress, enhance antioxidant activity, and modulate inflammatory pathways makes it a promising candidate for neuroprotection and overall neurological well-being [190].

##### General Conclusion

*Withania somnifera* emerges as a valuable botanical resource with significant neuroprotective effects. Its rich chemical composition, including bioactive compounds like withanolides, contributes to its diverse pharmacological properties, making it a potent agent for safeguarding against neurological disorders. From historical uses in traditional medicine to modern research validating its neuroprotective mechanisms, *Withania somnifera* stands out as a promising natural remedy for promoting brain health and combating neurodegenerative conditions.

#### 3.2.5. *Curcuma longa* (Turmeric)

##### Historical Background

*Curcuma longa*, commonly known as turmeric, is a member of the Zingiberaceae family and is recognized for its vibrant yellow color and distinct flavor. This rhizomatous herbaceous perennial plant has a long history of traditional medicinal use and is often referred to as the “golden spice” or “Indian saffron” due to its rich cultural significance [191]. One of the key bioactive compounds found in *Curcuma longa* is curcumin, which has been extensively studied for its various health benefits, including its neuroprotective effects [192]. Curcumin is known for its anti-inflammatory, antioxidant, and anticancer properties, making it a promising candidate for therapeutic applications [193].

##### Neuroprotective Chemical Metabolites

The chemical composition of *Curcuma longa*, particularly its curcuminoids, has been a subject of interest in research focusing on its therapeutic potential. Curcumin, the primary bioactive compound in *Curcuma longa*, has been shown to possess antioxidant properties and the ability to modulate various signaling pathways involved in inflammation and cell survival. These characteristics make curcumin a valuable component of *Curcuma longa* with diverse pharmacological effects, including neuroprotection [191].

##### Neuroprotective Properties

The neuroprotective effects of *Curcuma longa* are attributed to its ability to modulate various molecular pathways involved in neuronal health and function. Studies have shown that curcumin can attenuate inflammation, reduce oxidative stress, and inhibit the activation of transcription factors like NF-kappa B, which play a role in neurodegenerative processes. Additionally, curcumin has been found to upregulate the expression of heme oxygenase-1 and activate AMP-activated protein kinases, which are involved in cellular stress responses and energy metabolism, further contributing to its neuroprotective effects [192].

##### Clinical Applications

In the context of neurological benefits, *Curcuma longa* has shown promise in mitigating the effects of neurodegenerative diseases such as Alzheimer’s disease. Research indicates that the active compounds in *Curcuma longa*, particularly curcumin, can exert neuroprotective effects through multiple mechanisms, including the modulation of apoptotic signaling pathways and the enhancement of dopaminergic synaptic function. These findings suggest that *Curcuma longa* may have potential therapeutic applications in the prevention and treatment of neurodegenerative disorders [194].

Clinical studies have also explored the use of *Curcuma longa* in various neurological conditions. For instance, research has investigated the effects of *Curcuma longa* in status epilepticus induced by pilocarpine in rats, demonstrating its neuroprotective properties in mitigating seizure-induced neuronal damage [195]. Furthermore, *Curcuma longa* has been studied for its potential role in managing conditions like arthritis, where its anti-inflammatory properties may help alleviate symptoms and improve joint function [196].

In addition to its neuroprotective effects, *Curcuma longa* has been investigated for its role in other health conditions, such as diabetes and cardiovascular diseases. Studies have highlighted the cardioprotective effects of *Curcuma longa*, including its ability to regulate serum cardiac marker enzymes and exert antioxidant, anti-inflammatory, and hypolipidemic activities [197]. Moreover, research has demonstrated the potential of *Curcuma longa* in managing diabetes by reducing fasting blood sugar levels and HbA1C, indicating its anti-diabetic properties [198].

##### General Conclusion

Overall, the research on *Curcuma longa* supports its neuroprotective effects through various mechanisms, including anti-inflammatory, antioxidant, and anti-apoptotic pathways. The plant’s bioactive compounds, particularly curcumin, have shown promise in mitigating neurodegenerative processes and protecting neuronal health. Studies have provided insights into the potential applications of *Curcuma longa* in neurological conditions, highlighting its therapeutic benefits beyond traditional medicinal uses. Further research is warranted to explore the full potential of *Curcuma longa* in neuroprotection and its implications for human health.

**Figure 2:**
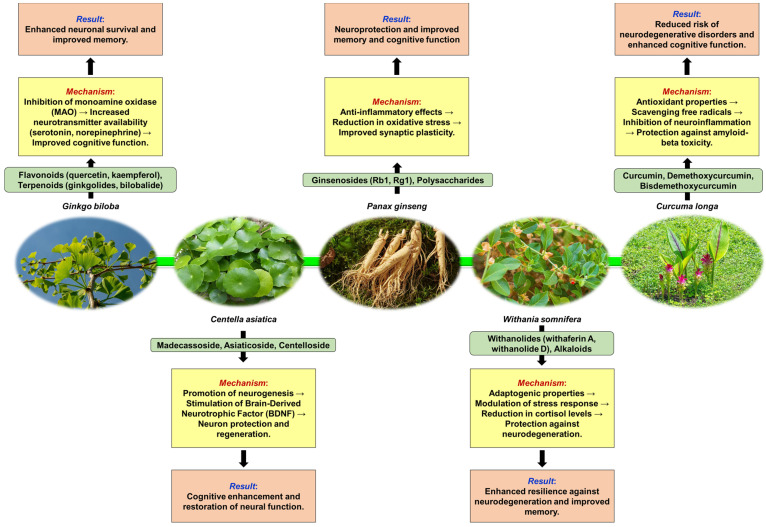
Mechanism of action of some neuroprotective plants: *Ginkgo biloba* (https://www.alamy.com/stock-photo/ginkgo-biloba.html?sortBy=relevant, accessed on 7 August 2024), *Centella asiatica* (https://www.alamy.com/stock-photo/centella-asiatica.html?sortBy=relevant, accessed on 7 August 2024), *Panax ginseng* (https://www.alamy.com/stock-photo/panax-ginseng.html?page=4&sortBy=relevant, accessed on 7 August 2024), *Withania somnifera* (https://www.alamy.com/stock-photo/Withania-somnifera.html?sortBy=relevant, accessed on 7 August 2024), and *Curcuma longa* (https://www.alamy.com/stock-photo/curcuma-longa.html?sortBy=relevant, accessed on 7 August 2024).

Figure 2 illustrates the mechanisms of action of various plants with neuroprotective properties. Each plant’s Latin name, major compounds, neuroprotective mechanism, and resulting neuroprotection are detailed. The figure provides an overview of how these plants exert their neuroprotective effects through specific compounds, ultimately leading to enhanced neuronal survival and function.

Table 3 presents a comprehensive overview of neuroprotective plants, detailing the utilized parts of these plants, the beneficial secondary metabolites or extract types, and the context of in vivo or in vitro studies conducted. It further highlights the effective dosages employed in these studies and the resultant outcomes observed. This synthesis of information underscores the diverse phytochemical constituents that contribute to neuroprotection, which have been shown to exert neuroprotective effects through various mechanisms. The data compiled herein may facilitate further investigation into the efficacy and safety of these natural products for clinical applications.

Table 4 presents the chemical structures of key secondary metabolites identified in various neuroprotective plants, presenting their structural diversity and highlighting their potential significance for neuroprotective properties.

## 4. Conclusions and Future Research Directions

In conclusion, this study on the impact of plants on the human brain has shed light on the intricate interplay between botanical species and brain health, highlighting the neuroprotective and neurotoxic potential of plants. The findings underscore the diverse mechanisms through which plant-derived compounds can influence neuronal function, oxidative stress, inflammation, and cognitive performance, offering promising avenues for neuroprotection and therapeutic intervention. Moving forward, future research directions in the field of plant-based neuroprotection and neurotoxicity should include the identification of novel neuroactive compounds, exploration of synergistic plant formulations, elucidation of molecular mechanisms of action, investigation of plant-rich diets, modulation of the gut–brain axis, targeting neuroinflammation and neuroplasticity, interaction with neurotransmitter systems, blood–brain barrier integrity, mitochondrial function, and epigenetic regulation. By advancing our understanding of the impact of plants on the human brain, researchers can harness the therapeutic potential of botanical elements to promote brain health, prevent neurological disorders, and enhance cognitive resilience.

## Figures and Tables

**Table 2:** Chemical structures and structural diversity of key secondary metabolites from neurotoxic plants. The chemical structures were created using KingDrawHD v1.4.5.-20230617 software.

Botanical Name of the Plant	Name of the Secondary Metabolites	Chemical Structures of the Metabolites	Structural Diversity
*Aconitum napellus*	Aconitine	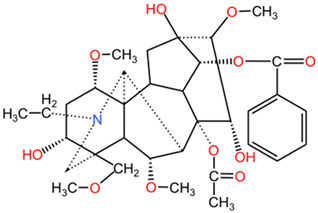	Structurally, aconitine (C_34_H_47_NO_11_) features a bicyclic skeleton with a long aliphatic chain, a methoxy group, and a hydroxyl group. The molecular architecture contributes to its cardiotoxicity and neurotoxicity.
	Mesaconitine	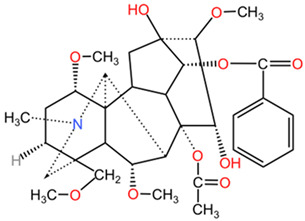	Mesaconitine (C_34_H_47_NO_10_) and hypaconitine (C_34_H_47_NO_9_) differ from aconitine through minor modifications in their functional groups and the presence of different hydroxy and methoxy groups, which impact their pharmacological profiles.
	Hypaconitine	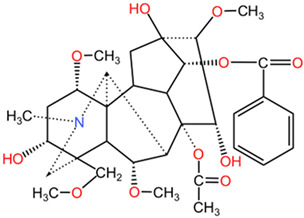	
	Benzoylaconine	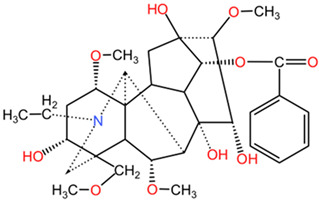	Benzoylaconine (C_32_H_45_NO_10_), benzoylmesaconine (C_31_H_43_NO_9_), and benzoylhypaconine (C_31_H_43_NO_10_) feature benzoyl group substitutions, which affect solubility and bioactivity. The incorporation of aromatic moieties tends to enhance their lipophilicity, potentially leading to improved cell membrane permeability.
	Benzoylmesaconine	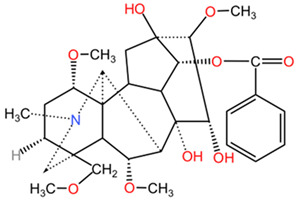	
	Benzoylhypaconine	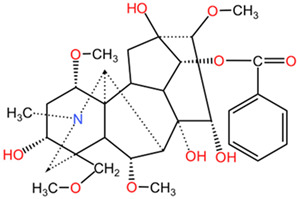	
*Atropa belladonna*	Hyoscyamine	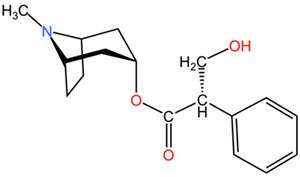	Hyoscyamine (C_17_H_23_NO_3_) is a naturally occurring tropane alkaloid, structurally characterized by a tropan ring fused with a cyclic ether. Its epimer, atropine (C_17_H_23_NO_3_), exhibits similar structural elements but differs in the spatial arrangement of its atoms, influencing its pharmacological efficacy and selectivity at muscarinic receptors.
	Atropine	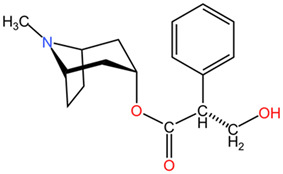	
	Scopolamine	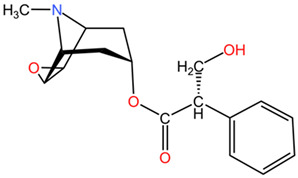	Scopolamine (C_17_H_21_NO_4_), another tropane derivative, features an additional hydroxyl group compared to hyoscyamine, imparting distinct central nervous system effects.
*Conium maculatum*	Coniine	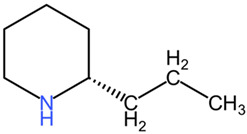	Coniine (C_8_H_17_N) and γ-coniceine (C_8_H_15_N) are piperidine derivatives, with structural simplicity relative to the previously mentioned compounds. Coniine consists of a saturated piperidine ring, while γ-coniceine has a double bond, influencing their activity and toxicity.
	γ-coniceine	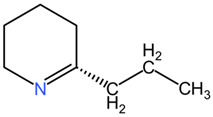	
*Oenanthe crocata*	Oenanthotoxin	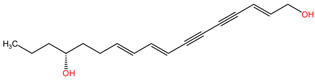	Oenanthotoxin and cicutoxin are structurally complex compounds that exhibit potent neurotoxic properties. Oenanthotoxin has a tricyclic structure with multiple functional groups, while cicutoxin contains an alkaloid structure with unsaturation, highlighting the versatility of alkaloid chemistry.
	Cicutoxin	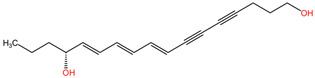	
*Ricinus communis*	Ricinine	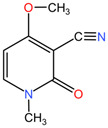	Ricinine (C_12_H_17_N_3_O_4_) differs significantly from the previous alkaloids, featuring a distinct tricyclic structure with nitrogen atoms in different orientations. Its diverse functional groups suggest varied biological activities compared to the other compounds.

**Table 3:** Overview of neuroprotective plants: used parts, beneficial secondary metabolites/extract type, in vivo/in vitro studies and effective dosage, and outcomes.

Plant, Family	Used Parts	Beneficial Secondary Metabolites/Extract Type	In Vivo/In Vitro Studies and Recommended Effective Dosage	Outcomes	Refs.
*Ginkgo biloba*, Ginkgoaceae	Leaves	EGb 761 extract (the standardized extract of dried leaves made with acetone 60% (*w*/*w*) as the extraction solvent): 22–27% flavonoids and 5–7% terpene lactones, consisting of 2.8–3.4% A, B and C ginkgolides and 2.6–3.2% bilobalide	Randomized, placebo-controlled trials: 240 mg/day (for 12–26 weeks), 2 × 120 mg/day (for 22–24 weeks)	Neuroprotection can be achieved through several mechanisms, which include the suppression of inflammation and apoptosis, a reduction in amyloid precursor protein levels, particularly Amyloid β, and the promotion of cell proliferation within the hippocampus. At the cellular level, the neuroprotective actions encompass the elimination of free radicals, enhancement of mitochondrial performance, a decrease in blood viscosity, modulation of serotonin concentrations across different brain regions, and an elevation of dopamine levels in the prefrontal cortex. These processes collectively contribute to the preservation of neuronal health and function.	[199,200]
			Mice, rats: 50–100 mg/kg/day for 14 days–7 months	Antistress effects, decreasing anxiety level and MAO activity.	[201]
			Mice: 10 mg/kg/day (i.p.) for 17 days	Reduction in lipid peroxidation and superoxide radical production, and modulation of serotonergic and dopaminergic neurotransmission → antidepressant-like effect.	[201]
			Randomized, placebo-controlled clinical trials evaluating efficacy in dementia: 120 mg/day (52–26 weeks), 240 mg/day (12–24 weeks)	Significant improvement in cognitive function.	[199,201]
			Rats: 4 mg/kg BW (for 8 weeks)	The extract promotes the secretion of estrogen-dependent biogenic amines within the hippocampus. Additionally, it mitigates neuronal damage in the hippocampus through its antioxidant properties and its ability to stimulate adiponectin release. Furthermore, it has the potential to manage memory and learning impairments effectively.	[202]
			Healthy volunteers (18–26 years): 120, 240, 360 mg/day—single doses; 120 mg/day for 6 weeks	Improved cognitive performance, self-esteem, and mood, increasing attention efficiency and improving memory quality.	[199]
			Patients with mild to moderate dementia, Alzheimer’s disease (50–70 years): 240 mg/day for 24 weeks	Overall improvement in cognitive functions and general condition.	[199]
*Centella asiatica*, Apiaceae	The aerial parts (leaves and stems)	70% hydro-ethanolic extract	Clinical study (patients 18–60 years old): 500 mg/capsule, twice daily for 60 days	Overall improvement in stress, anxiety, depression, adjustment, and attention.	[203]
		Ethanolic extract	Rats: 200, 300 mg/kg BW/day (p.o.) for 21 days	Improved neurobehavioral activity and reduced tissue death due to lack of oxygen. Additionally, there was considerable safeguarding against neuronal damage, as indicated by the preservation of hippocampal and cortical neurons.	[204]
		Aqueous leaf extract	Rats: 500 mg/kg BW (p.o.)	The extract reduced behavioral impairments and enhanced learning and memory performance.	[205]
		ECa233 (standardized extract): 85% triterpenoid glycoside, divided into madecassoside (53.1%) and asiaticoside (32.3%) in a ratio of 1.5:0.5; 15% triterpenoid saponins, of which less than 1% are triterpenic acid metabolites (madecassic acid and asiatic acid)	Rats: 30 mg/kg (p.o.)	Significantly enhanced memory retention, with an increase in synaptic-related proteins in the hippocampus, leading to a strong synaptic plasticity enhancement. It also demonstrated an anxiolytic effect.	[206,207]
			In vitro (neuroblastoma cell lines): 100 μg/mL	Significant stimulatory effects on the elongation of neuroblastoma cell neurites.	[208]
		Extract containing tannic acid (29.9 mg/g), asiaticoside (1.09 mg/g), and asiatic acid (48.89 mg/g)	Randomized, double-blind, placebo-controlled clinical study (mean age: 65 years): 250, 500, 750 mg/day for 2 months	Enhanced overall cognitive functions, memory, and self-rated mood.	[209]
			Clinical study (mean age: 65 years): 1000 mg/day for 6 months	Significant cognitive improvement.	
		Capsules of 70% hydro-ethanolic extract of dried aerial parts	Open-label (adults with generalized anxiety disorder, mean age 33 years): 500 mg twice daily for 2 months	The capsules demonstrated a multifaceted impact on psychological well-being, as they significantly lowered anxiety levels, increased self-perceived stress, decreased the depression index, enhanced the adjustment index, and elevated attention levels.	[203]
*Panax ginseng*, Araliaceae	Roots	*Panax ginseng* extract: ginsenosides (the main, 70–80%, found in fresh ginseng are Rb2, Rb1, Re, Rg1, Rc)	Rats: 100 mg/kg (p.o.) for 2 weeks	Enhanced alterations in the midbrain and striatum, as well as demonstrated a partial therapeutic effect in a rat model of Parkinson’s disease.	[179,210]
		Total saponins	Mice: 50, 100, 200 mg/kg/day (p.o.)	Memory deterioration was mitigated through the enhancement of antioxidant levels in the hippocampus, alongside an increase in proteins associated with neural plasticity.	[211]
		Glycoprotein PGL-1	Rats: 40, 80, 160 mg/kg·d^−1^ (i.p.) for 35 days	The treatment significantly improved the learning and memory abilities in the Alzheimer’s disease rat model. The neuroprotective effects of PGL-1 may be associated with its ability to suppress nitric oxide synthesis.	[212]
		Ginsenoside Rg1	Mice: 10 mg/kg (i.p.) for 14 days	Ginsenoside Rg1 has demonstrated the ability to inhibit the activation of microglial cells triggered by lipopolysaccharides and to safeguard dopaminergic neurons within the nigrostriatal pathway. This suggests that Rg1 could serve as a potential therapeutic approach to mitigate inflammatory harm associated with chronic neurodegenerative disorders.	[213]
		Korean red ginseng (KRG)	Rats: 20, 50, 100 mg/kg (i.p.) for 14 days	The administration of KRG has been shown to enhance cognitive functions and capabilities, including the processes of learning acquisition, memory consolidation, and the reduction in fear memory in the animal model exhibiting memory impairment due to SPS. These results indicate that KRG may serve as a beneficial alternative therapy for cognitive dysfunctions associated with traumatic stress, particularly in conditions like PTSD.	[214]
*Withania somnifera*, Solanaceae	Roots, leaves (occasionally)	A standardized extract: 2.6% withanolides including withanolides IV (0.87%) and V (0.65%), withaferin A (0.56%), withanolide A (0.20%) and B (0.06%), and 12-deoxy withastramonolide (0.26%)	In vitro (human neuroblastoma cell line): 12–50 µg/mL	The extract demonstrated neuroprotective activities through multiple mechanisms, including protection against Aβ and acrolein toxicity, reduction in oxidative stress, and inhibition of AChE activity.	[215]
		Withanone extracted from the roots	Rats: 5, 10, 20 mg/kg (p.o.) for 21 days in vitro (PC-12 cell line)	Withanone exhibits a significant protective effect against amyloid β toxicity, which is crucial in the context of neurodegenerative diseases such as Alzheimer’s. Withanone not only enhances memory retention but also significantly mitigates cognitive impairment associated with Aβ accumulation. Furthermore, the compound effectively attenuates elevated levels of pro-inflammatory cytokines, suggesting a dual mechanism of action that involves both neuroprotection and anti-inflammatory effects.	[216]
		Ethanolic extract	Rats: 200 mg/kg (p.o.) for 30 days	The extract exhibits properties that combat neurotoxicity through its antioxidant and anti-inflammatory actions, effectively mitigating oxidative stress, neuroinflammation, and excitotoxicity. Furthermore, it may help preserve cholinergic function by regulating acetylcholinesterase activity, suggesting its potential role as a cognitive enhancer. Consequently, ashwagandha extract could be considered a complementary treatment option for Alzheimer’s disease.	[217]
		Hydro-ethanolic extract from roots	Rats: 500 mg/mL (p.o.) for 6 weeks	The extract may safeguard against memory deficits associated with PTSD, both in the short and long term, potentially by counteracting oxidative stress in the hippocampus.	[218]
		Standardized ethanolic extract: 11.90% withanolide glycosides, 1.05% withaferin A, 40.25% oligosaccharides, 0.05% alkaloids, 3.44% polysaccharides.	Double-blind, randomized, placebo-controlled study (18–60 years): 12, 250 mg; 1000 mg/individual; 60 days	The administration of ashwagandha was associated with a decrease in subjective experiences of stress and anxiety in chronically stressed adults. Furthermore, it has been shown to lower serum levels of cortisol and C-reactive protein, as well as reduce pulse rate and blood pressure. Additionally, ashwagandha supplementation contributes to improvements in fasting blood glucose levels and lipid profiles. These findings suggest that ashwagandha may play a beneficial role in managing physiological and psychological stress responses, alongside enhancing metabolic health indicators.	[219,220]
*Curcuma longa*, Zingiberaceae	Rhizome	Curcumin	Rats: divided into two different groups: Group 1: rats were given only glucose solution Group 2: rats were treated with 25% alcohol for one month, then divided into two subgroups: (a) those who continued treatment with 25% alcohol only (b) those who received treatment with curcumin 80 mg/kg body combined with 25% ethanol	Alcohol treatment in the rats resulted in significant increases in cholesterol, phospholipids, free fatty acids, and TBARS levels in the brain compared to the control group, which received no alcohol at all. It was shown that administration of the alcoholic extract with curcumin led to a considerable decrease in cholesterol, phospholipids, and TBARS levels, and glutathione levels were partially restored. This supports the fact that curcumin has a protective effect on the brain, reducing oxidative stress and alleviating alcohol damage.	[221]
		Alcoholic extract dissolved in Tween 20 before administration	Mice: 100 mg/kg (p.o.) for 13 weeks	Curcumin administration was effective in significantly improving memory, demonstrating once again its neuroprotective effect.	[222]
		Curcumin	In vitro (rat adrenal pheochromocytoma PC12 cells): 0, 25, 50, 100, 150 and 200 μM for 24 h	In vitro treatment with curcumin has been shown to significantly reduce cellular damage and decrease 6-OHDA-induced mortality.	[223]
		Aqueous extract	Rats: 100 mg/kg BW/day	Results of the in vivo study showed that *Curcuma longa* extract helped to improve learning ability in elderly rats, having a neuroprotective effect against cognitive impairment associated with aging or Alzheimer’s disease.	[224]

p.o. = per os; s.c. = subcutaneously; i.p. = intraperitoneally; i.v. = intravenously.

**Table 4:** Chemical structures and structural diversity of key secondary metabolites from neuroprotective plants. The chemical structures were created using KingDrawHD v1.4.5.-20230617 software.

Botanical Name of the Plant	Name of the Secondary Metabolites	Chemical Structures of the Metabolites	Structural Diversity
*Ginkgo biloba*	Flavonoids (kaempferol, quercetin, isorhamnetin derivatives).	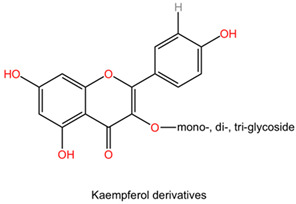	Flavonoids are polyphenolic compounds characterized by a flavone backbone, featuring various substitutions that alter their chemical properties and biological activities. Kaempferol has multiple hydroxyl groups that can donate hydrogen and provide antioxidant properties. Quercetin possesses a similar structure but has an additional double bond and ketone, enhancing its bioactivity and solubility. Isorhamnetin is a methoxy derivative of quercetin that exhibits different pharmacokinetics and increased lipid solubility.
		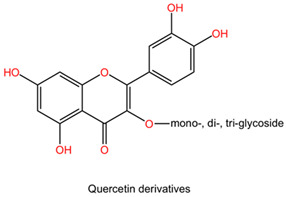	
		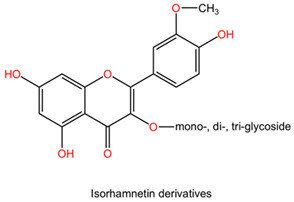	
	Terpene lactones: ginkgolides (A, B, C, J, M) and bilobalide	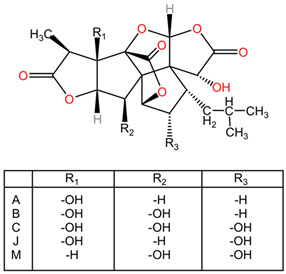 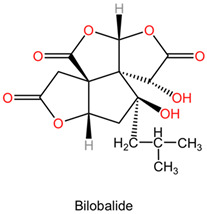	Ginkgolides are a class of diterpene lactones found in *Ginkgo biloba*, exhibiting significant pharmacological properties. The major ginkgolides—A, B, C, J, and M—possess distinct structural features, including two terpene units and various functional groups that diversify their activity. Ginkgolide A and B are characterized by their unique bicyclic structures, impacting their receptor binding interactions. Bilobalide, another key compound, is a sesquiterpene derivative that complements the activity of ginkgolides.
*Centella asiatica*	Triterpenoid glycoside: madecassoside, asiaticoside Triterpenoid saponins: madecassic acid, asiatic acid	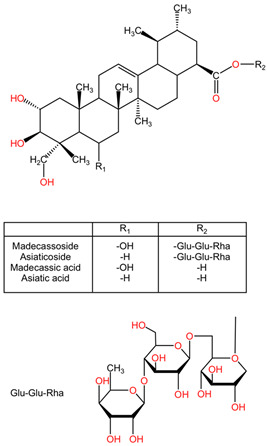	Triterpenoid glycosides, such as madecassoside and asiaticoside, display a complex structure consisting of a triterpene backbone linked to sugar moieties. This glycosylation affects their solubility and bioavailability. Madecassoside exhibits a pentacyclic triterpene structure with various sugar attachments, increasing its medicinal properties. Asiaticoside, a closely related compound, differs from its sugar moieties, which contributes to its distinct pharmacological profile. Triterpenoid saponins, such as madecassic acid and asiatic acid, showcase a steroid-like structure that differs in functional group orientation and substitution patterns.
*Panax ginseng*	Ginsenosides (Rg1, Re, Rb1, Rc, Rd)	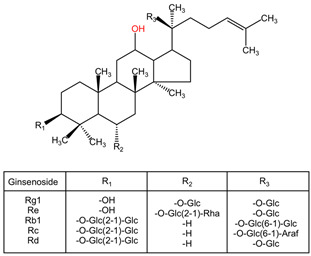	Ginsenosides encompass a diverse range of triterpenoid saponins, including Rg1, Re, Rb1, Rc, and Rd. Each ginsenoside exhibits variability in sugar chains and structure, influencing its pharmacological properties. Rg1 has a protopanaxadiol framework that promotes nerve cell regeneration. Re and Rb1 contain additional sugar moieties that enhance their bioavailability and activity in modulating immune responses.
*Withania somnifera*	Withanolide A	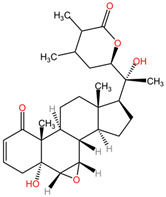	Withanolides, derived from *Withania somnifera*, encompass a diverse group of steroidal lactones. Important representatives, including withanolide A, B, withanone, and withaferin A, exhibit variations in side chains and functional groups. Withanolide A features a complex cyclic structure with multiple hydroxyl substitutions, enhancing its bioactivity. Withaferin A has a highly oxygenated ring structure that contributes to its potent cytotoxicity against cancer cells.
	Withanolide B	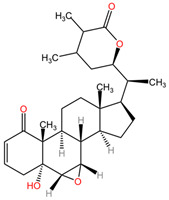	
	Withanone	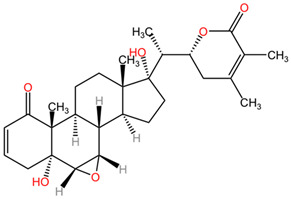	
	Withaferin A	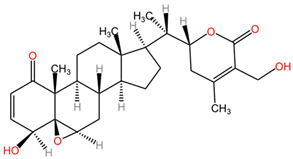	
	Withanoside IV	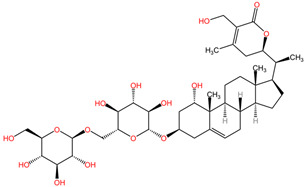	
	Withanoside V	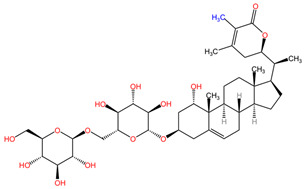	
*Curcuma longa*	Curcumin	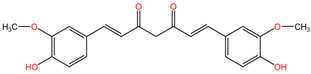	Curcumin (C_21_H_20_O_6_) features a unique structure characterized by two feruloyl moieties connected by a methylene bridge. This structure is pivotal to curcumin’s interaction with biological targets.
	Demethoxycurcumin	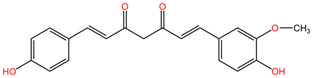	Demethoxycurcumin (C_20_H_18_O_5_) is a derivative where one of the methoxy groups from curcumin is removed. This structural alteration has been shown to affect its binding affinity to various biological targets and its resultant therapeutic potential.
	Bisdemethoxycurcumin	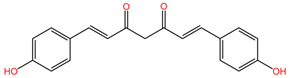	Bisdemethoxycurcumin (C_19_H_16_O_4_) has both methoxy groups removed from the parent curcumin structure. This compound exhibits altered biological activities, showcasing how subtle changes in structure can lead to significant variations in pharmacodynamics.

## Data Availability

There are no additional data to be published.
